# Performance of a Self-Paced Brain Computer Interface on Data Contaminated with Eye-Movement Artifacts and on Data Recorded in a Subsequent Session

**DOI:** 10.1155/2008/749204

**Published:** 2008-05-11

**Authors:** Mehrdad Fatourechi, Rabab K. Ward, Gary E. Birch

**Affiliations:** ^1^Image and Signal Processing Laboratory, Department of Electrical and Computer Engineering, University of British Columbia, Vancouver, BC, Canada V6T 1Z4; ^2^Institute for Computing, Information and Cognitive Systems, University of British Columbia, Vancouver, BC, Canada V6T 1Z4; ^3^Neil Squire Society, Burnaby, BC, Canada V5M 3Z3

## Abstract

The performance of a specific self-paced BCI (SBCI) is investigated using two different datasets to determine its suitability for using online: (1) data contaminated with large-amplitude eye movements, and (2) data recorded in a session subsequent to the original sessions used to design the system. No part of the data was rejected in the subsequent session. Therefore, this dataset can be regarded as a “pseudo-online” test set. The SBCI under investigation uses features extracted from three specific neurological phenomena. Each of these neurological phenomena belongs to a different frequency band. Since many prominent artifacts are either of mostly low-frequency (e.g., eye movements) or mostly high-frequency nature (e.g., muscle movements), it is expected that the system shows a fairly robust performance over artifact-contaminated data. Analysis of the data of four participants using epochs contaminated with large-amplitude eye-movement artifacts shows that the system's performance deteriorates only slightly. Furthermore, the system's performance during the session subsequent to the original sessions remained largely the same as in the original sessions for three out of the four participants. This moderate drop in performance can be considered tolerable, since allowing artifact-contaminated data to be used as inputs makes the system available for users at ALL times.

## 1. Introduction

A self-paced brain computer interface (SBCI) allows individuals to control devices using their brain signals only, whenever they wish [1]. The performance of SBCI systems is usually determined via two objective functions: (1) the true positive (TP) rate, that is, the percentage of intentional control (IC) commands that are correctly detected by the SBCI system, and (2) the false positive rate (FP), that is, the rate of false positives generated by the system during the periods for which the user does not intend control (no control (NC) periods). In other words, the FP rate is calculated as the percentage of false decisions in the NC periods.

The above functions, however, have traditionally been calculated over periods not contaminated with artifacts. Since physiological artifacts such as eye movement (EOG) and muscle movements (EMG) may frequently occur during testing of an SBCI system, they need to be handled efficiently. Various automatic rejection and removal techniques have been proposed in the literature to deal with these artifacts, however, most of them suffer from some shortcomings that may limit their application during online testing of the system [2].

One solution is to design an SBCI system whose structure is robust in the presence of artifacts. As an example, consider an SBCI system that uses features extracted from different neurological phenomena, each belonging to a different frequency band. We postulate that such an SBCI system should have a good performance in the presence of artifacts, since each type of artifact is usually prominent only over specific frequency bands. For example, EOG artifacts are mostly of a low-frequency nature, while EMG artifacts are mostly of a high-frequency nature. To test this hypothesis, we examine the performance of the system proposed in [3] using data contaminated with artifacts. This SBCI system uses features extracted from three neurological phenomena, each belonging to a different frequency band: (1) movement-related potentials (MRPs), (2) changes in the power of Mu rhythms (CPMR), and (3) changes in the power of Beta rhythms (CPBR). These phenomena are used to distinguish between intentional control (IC) commands and no control (NC) EEG segments.

In [3], the performance of the system was tested using artifact-free data from four able-bodied participants. The data were collected over five sessions, and data that were not contaminated with large-amplitude eye-movement artifacts were used to train the system. Using these data, an average TP rate of 56.2% was achieved while the average FP rate was 0.1%.

We conducted two studies to test the performance of the system during periods contaminated with artifacts. In the first study, we evaluated the performance of the system over the epochs from the first five sessions that are contaminated with large-amplitude eye movement activities (e.g., eye blinks). In the second study, we evaluated the performance of the system on data recorded in a “subsequent session”. For each participant, a sixth session, “the subsequent session” was recorded at a later date, which took place one to six days after the fifth session. The same paradigm as in the previous 5 sessions was used for data recording. All epochs, whether or not contaminated with ANY artifact, were used in the second study. This study can be regarded as a performance test of the system in a “pseudo-online” environment, but where no feedback about the user 's performance is given to the user.

In the next section, we provide more background information about the state of the art of SBCI systems, methods of handling artifacts, and how using more than one neurological phenomenon could lead to a more robust performance in the presence of artifacts.

## 2. Background

### 2.1. State of the Art of SBCI Systems

**Figure 1:**
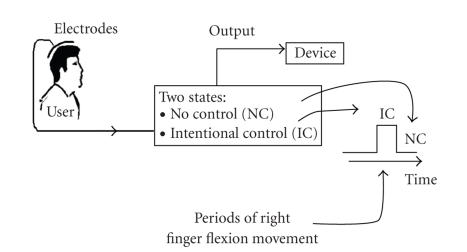
A typical SBCI system that identifies an IC command related to the execution of right finger flexion.

Currently, two different approaches for the design of BCI systems are pursued: synchronized and self-paced [4]. In the synchronized approach, which forms the traditional approach to the design of BCI systems, the user can only perform the control in certain time intervals that are specified by the system. While synchronized BCI systems can achieve high classification accuracy (>90%), their application is somewhat limited, because the user cannot perform the control at all times. Moreover, most synchronized systems assume that the user will exert an intentional control (IC) command during known prespecified control periods. In other words, synchronized BCI systems do not recognize those periods over which the user does not wish to perform a control action (called no control, NC, periods). As a result, these systems may become unstable during NC periods [5]. To address these shortcomings of synchronized BCI systems, the concept of a self-paced BCI (SBCI) has been proposed An SBCI system is constantly available for use, thus it should be able to identify IC patterns from the NC periods. Figure 1 shows a typical example of a 2-state SBCI system designed to recognize IC patterns (generated as the result of a right finger flexion) from the NC states. When the system detects an IC command, it generates a logical state “1”, otherwise, the output is the logical state “0”.

It is difficult to compare the results obtained from different SBCI studies directly with each other, because the protocols for conducting the experiments, the gathering of IC and NC epochs, the evaluation of the performance as well as the neurological phenomena used vary significantly amongst the studies. For this reason, most SBCI papers simply compare the current results with their previous work. In Table 1, we report a summary of the results obtained in various EEG-based SBCI studies. Keeping in mind that a direct comparison is not possible, this table only roughly hints at the relative performance of different SBCI systems. The rows of this table show different SBCI studies. The columns 3 to 6 show the rate at which the system generates an output, true positive rate (TPR), false positive rate (FPR), and the false activation rate (FAR), respectively. FAR shows how many false positives on average are expected in every 100 seconds of data. Please note that studies 1, 2, and 3 report the TPR values for high FPR values using the receiver operating characteristic (ROC) curve. However, in order to make a fair comparison, the TPR values in Table 1 are estimated from the same ROC plots for low FPR values.

As seen from Table 1, the first five studies generated relatively high numbers of false positives. The only system that had a low FP rate was Study 6 [3], the performance of which was investigated under new conditions in the current study. Please note that in [3] only those epochs that were not contaminated with large-amplitude eye movement activities (e.g., eye-blink artifacts) were used for evaluating the performance. Since this system showed a good performance in terms of having low FP rates while having reasonable TP rates, the next logical step is to test the performance over data that are contaminated with physiological artifacts.

### 2.2. Physiological Artifacts in BCI Systems

Physiological artifacts are undesired potentials that contaminate EEG signals. These artifacts can modify the shape of a neurological phenomenon that drives a BCI system. As a result, artifacts may prevent an SBCI system from correctly recognizing a control command, or they may cause the system to identify an artifact-related pattern as an intentional control command (resulting in a false activation). The two physiological artifacts that have been examined the most in BCI studies are electrooculogram (EOG) and electromyogram (EMG) artifacts. A number of studies have shown that EOG and EMG activities may generate artifacts that affect the neurological phenomena used in a BCI [11, 12]. These artifacts are often involuntary, and controlling them during signal acquisition is not easy. Therefore, there is a need to avoid, reject, or remove them from EEG signals.

The results of a recent review have pointed out that most BCI studies do not report on how they handle the presence of artifacts in their proposed design or they manually reject prominent artifacts [2]. These systems, however, may still encounter problems during online testing of the system, where physiological artifacts occur frequently. In some studies, automatic methods are used to reject data contaminated with artifacts [6, 13–15]. One shortcoming of automatically rejecting the data epochs that are contaminated with artifacts is that the periods for which the system is available for control greatly decreases. For example, physiological artifacts such as eye blinks happen frequently, making the SBCI system unavailable for control during those periods.

An alternative method is to employ automatic artifact removal methods such as filtering, regression, and independent component analysis (see [2] for a detailed review). Few BCI studies have used these approaches for removing artifacts [16–18]. Unfortunately, several problems arise when these automated artifact removal methods are used. Firstly, most artifact removal methods such as filtering and regression may remove useful information related to the neurological phenomena from the EEG signals. Although sophisticated methods such as independent component analysis reduce the amount of removed brain activity, this problem is not completely alleviated [19]. Secondly, removing muscle (EMG) artifacts is not a straightforward process, as various sources of EMG artifacts should be identified. The third problem is the computational complexity of the artifact removal methods, which is much higher than in artifact rejection methods.

An alternative method to these approaches is discussed in the next subsection.

### 2.3. Robustness in the Presence of Artifacts

An alternative approach to removal or rejection of artifacts from EEG signals is to design a BCI system that is robust in the presence of artifacts. In that case, the presence of artifacts would not affect the performance of the BCI system. This approach has not received much attention in the BCI literature. If this approach is successfully implemented, it would have several advantages over the previous approaches. First, the system becomes available for use at all times. Second, neurological phenomena would not be removed from the brain signals, Third, if this design deals with all the artifacts together, then there would be no need to design separate methods to deal with different types of EOG artifacts (e.g. eye blinks, saccades, eye rolling) as well as EMG artifacts (e.g., moving facial muscles, jaw clenching).

An approach to increase the robustness of an SBCI in the presence of artifacts is to design the system that uses more than one neurological phenomenon to detect IC commands. Every neurological phenomenon is known to have its own spatiotemporal characteristics that are more prominent in a particular frequency band. A system that depends on a certain neurological phenomenon is expected to be robust to artifacts whose frequency contents are concentrated in frequency bands other than that of the neurological phenomenon. As an example, movement-related potentials (MRPs) have low-frequency contents <4 Hz [6], while muscle artifacts mostly lie within frequency bands >10 Hz [11, 20]. Thus, an MRP-based BCI system is expected to have a robust performance in the presence of muscle artifacts. Similarly, the Mu and Beta rhythms cover frequencies above 8 Hz, while eye-movement artifacts such as eye blinks mostly affect lower-frequency components of EEG signals. As a result, BCI systems based on the Mu and Beta rhythms are expected to have robust performance in the presence of EOG artifacts.

**Figure 2:**
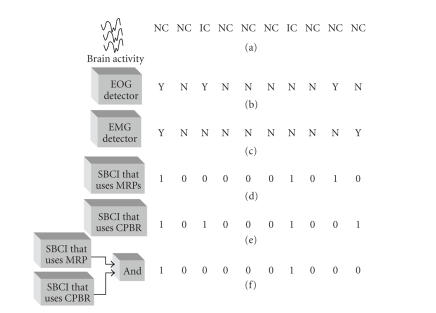
A demonstration of how using more than one neurological phenomenon can lead to improvement in the performance. (a) Brain states of the user (NC: no control; IC: intentional control), (b) the output of the “ideal” EOG detector (N: no artifact; Y: artifact-contaminated), (c) the output of the “ideal” EMG detector, (d) the output of the MRP-based SBCI (“1”: IC detected; “0”: NC detected), (e) the output of the CPBR-based SBCI, and (f) the output of the Hybrid BCI system.

Now consider an SBCI system that at any instance of time uses features extracted from MRPs as well as changes in the power of Mu and Beta rhythms. If the three feature vectors extracted from these three neurological phenomena are classified separately, this system is expected to be more robust to the presence of muscle movement (EMG) and eye movement (EOG) artifacts than an SBCI system that uses features extracted from only one neurological phenomenon. This is because the performance of the system is affected only partially by the presence of EOG and EMG artifacts. This idea is explained in a simplified form in Figure 2.

Consider the hypothetical case where an SBCI system is able to identify all of the input patterns correctly, as long as no artifact is present in the input signal. Figure 2(a) shows the true user's brain state over a short period of time. To design this simple SBCI system, we assume that the brain has two states only: intentional control (IC) and no control (NC). Suppose that during the period shown in Figure 2, the user issues only two IC commands as shown in Figure 2(a).Figure 2(b) shows the output of an ideal eye movement (EOG) detector that can correctly identify all EOG artifacts. Figure 2(c) shows the output of an ideal muscle movement (EMG) detector. The letter “Y” shows the period that is contaminated with the specific artifact, and the letter “N” shows the period that is not contaminated with this artifact. Figures 2(d) and 2(e) show the outputs of two SBCI systems that use MRP and CPBR, respectively. Here, “1” is the logical output generated by the system when an “IC” state is detected, and “0” is the logical output when an “NC” state is detected. Figure 2(f) shows the output of an SBCI system that combines the outputs of the SBCIs shown in Figures 2(d) and 2(e), using the logical “AND” operator.

Since EOG artifacts mostly affect the low-frequency potentials such as MRPs, it is expected that the performance of the MRP-based SBCI system is mostly affected by these artifacts. As the first IC command coincides with the presence of an EOG artifact, the system does not detect this IC command (see Figure 2(d)). Similarly, the SBCI system in Figure 2(e) uses features extracted from the relatively high-frequency CPBR, so its performance is vulnerable in the presence of high-frequency EMG artifacts. This system correctly identifies both IC commands; however, two false positives occur in the NC periods due to the presence of EMG artifacts. Since the output of the system in Figure 2(f) combines the logical outputs of Figures 2(d) and 2(e) using the “AND” operator, it would only detect an “IC” when both the MRP-based and the CPBR-based SBCI systems detect an “IC”. As a result, the performance of this system in detecting IC commands is inferior. However, because of the “AND” operator, the number of false positives is significantly lower. Since *p*(NC) ≫ *p*(IC), it is expected that the system in Figure 2(f) will have a superior performance compared with the other two systems during the NC periods (see [3] for the mathematical proof). The same argument can also be applied for artifact-contaminated periods. Since EOG artifacts affect MRPs more than CPBR, it is expected that the system in Figure 2(f) will have a robust performance in their presence. A similar argument can be made about EMG artifacts, which mostly affect the higher frequency neurological phenomena such as CPBR. Note that when EOG and EMG artifacts occur simultaneously (see the first time sample in Figure 2), then the performance of all three systems may be affected. As can be seen, the system in Figure 2(f) has a TP rate of 50%. However, it has only one false positive, which is lower than that the number generated by the other two systems.

In the next section, an overall description of the SBCI system proposed in [3] is provided, and the data collection procedure and evaluation method used are discussed briefly.

## 3. Methods

### 3.1. Self-Paced Brain Computer Interface Design

**Figure 3:**
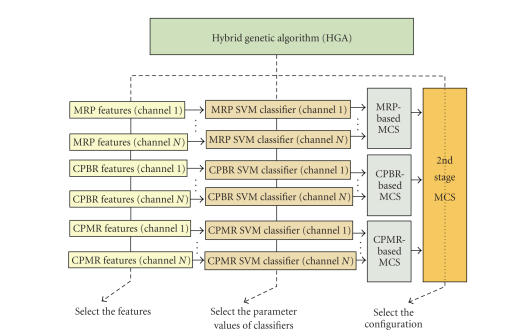
The overall structure of the improved SBCI.

The structure of the SBCI is shown in Figure 3. The system uses features extracted from *N* = 18 bipolar EEG signals. For each neurological phenomenon and for every EEG signal, features are extracted using a stationary wavelet transform (SWT) with a 5-level decomposition. As for the Mu and Beta features, these frequency bands are first bandpass filtered, and the sample values are squared so that power values are calculated. The SWT is applied then to calculate the wavelet coefficients. To reduce the dimensionality of the wavelet feature space, matched filtering (using the cross-covariance function) with a template is performed. The template is created for each neurological phenomenon and for each EEG channel by averaging the data in the training epochs. After calculating the cross-covariance for each epoch, the features representing the maximum of the cross-correlogram over a period of 0.125 second as well as the time this maximum occurred are extracted. This process is only carried out for the lowest approximation and detail level of the SWT decomposition. For each neurological phenomenon and for each EEG channel, a support vector machine (SVM) is designed (resulting in a total of 3*N* classifiers). The output of each SVM is a logical state “1” when an IC pattern is detected and is “0” in other cases. For each neurological phenomenon, a multiple classifier system (MCS) classifies the outputs of *N* SVMs using the majority voting rule. A 2nd-stage MCS combines the outputs of the three MCSs (each MCS is attributed to one neurological phenomenon) to generate the final classification label. A hybrid genetic algorithm (HGA) is then employed. This algorithm simultaneously finds (1) the subset of features, (2) the parameter values for each SVM, and (3) the configuration of combining the three MCSs that leads to near optimal performance (measured as the TPR/FPR ratio). Please see [3] for more details on the method of training and model selection of this SBCI system.

### 3.2. Data Collection

Data were collected from four right-handed (three males and one female) able-bodied participants between 31 and 56 years old. They had all signed consent forms prior to participation in the experiment.

Intentional control (IC) data were collected as participants performed a guided right index finger flexion movement. At random intervals of 5.6 to 7.0 seconds (mean of 6.7 seconds),a white circle of 2 cm diameter was displayed on the user's monitor for 1/4 second, prompting the participant to perform a movement by pressing a switch. In response to this cue, the subject had to perform a right index finger flexion one second after the cue appeared The 1-second delay was used to avoid visual evoked potential (VEP) effects caused by the cue. This is the time that the individual is expected to attempt the movement, but this time may vary from one person to another and from one movement attempt to another (see [21] for more details).

EEG signals were recorded from 13 monopolar EEG channels (according to the International 10–20 system at F_1_, F_*z*_, F_2_, FC_3_, FC_1_, FC_*z*_, FC_2_, FC_4_, C_3_, C_1_, C_*z*_, C_2_, and C_4_ locations) and were sampled at 128 Hz. The signals were then converted to bipolar EEG signals since such electrodes are more likely to generate more discriminant MRP features than monopolar electrodes [6]. The conversion was carried out by calculating the difference between adjacent EEG channels and resulted in the generation of the following 18 bipolar EEG: F_1_-FC_1_, F_1_-F_*z*_, F_2_-F_*z*_, F_2_-FC_2_, FC_3_-FC_1_, FC_3_-C_3_, FC_1_-FC_*z*_, FC_1_-C_1_, FC_*z*_-FC_2_, C_1_-C_*z*_, C_2_-C_4_, FC_2_-FC_4_, FC_4_-C_4_, FC_2_-C_2_, FC_*z*_-C_*z*_, C_3_-C_1_, C_*z*_-C_2_, and F_*z*_-FC_*z*_.

During the experiment, the participants performed a right index finger flexion. A threshold-based detector was used to mark any epoch with EOG amplitude above 25 *μ* as “an epoch contaminated with eye-movement artifacts”. The amplitude was measured as the difference between two electrodes, one placed at the eye level and the other below the right eye. Epochs of the IC type consisted of artifact-free data collected over an interval containing the onset of movement (measured as the finger switch activation).The interval started at *t*_start_ = −1 second, that is, 1 second before the onset of movement and ended at *t*_finish_ = 1, that is, 1 second after the onset of movement. Table 2 shows the timetable of recording the data for all participants. For each participant, “Day 1” was considered as the origin date, and the dates, when the rest of the data were collected, were numbered relative to “Day1”.

An SBCI should differentiate between IC and NC epochs. For this reason, data in NC sessions are also needed to represent the epochs during which the user did NOT intend to control. During an NC session, participants were asked to count the number of times that a white ball bounced off the monitor's screen. The NC sessions thus contained attentive as well as nonattentive NC data. Each NC session lasted approximately two minutes. During each recording day, up to two such NC sessions were recorded. The NC segments were selected as follows: a window of width (*t*_finish_ − *t*_start_) seconds was slid over each EEG signal collected during an NC session by a step of 16 time samples (0.1250 second). For each NC epoch, features were extracted and classified by the SBCI. This resulted in 8 classification decisions per second by the system. For each 1-second window where artifacts were not detected, features were extracted for training the SBCI system.

The IC and NC epochs for which the EOG activity exceeded a predefined threshold (±25 *μ*V) were marked as contaminated with large-amplitude eye-movement artifacts and were not used in the training of the system.

### 3.3. Evaluation

In [3], a five-fold nested cross-validation was used to evaluate the performance of the system on the so-called “artifact-free” data. The inner cross-validation set was used for model selection, and the outer cross-validation set was used to estimate the generalization error. For each outer cross-validation set, 20% of the data were used for testing and the rest were used for training and model validation. In order to select the models, the datasets were further divided into five folds. For each fold, 80% of the data were used for training the classifier, and 20% were used for model validation. To test the performance of the system on artifact-contaminated data, all the epochs which are marked as artifact-contaminated were used as the test set in Study 1.

**Figure 4:**
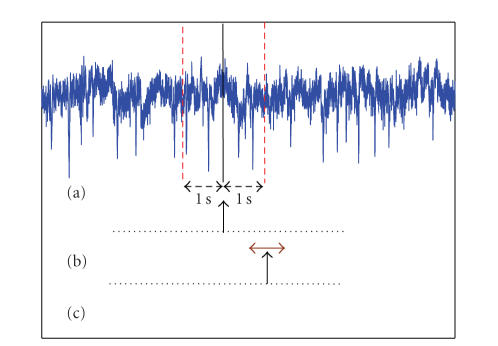
Method of calculating the TP rate: (a) EEG signal, (b) the output of the hand switch, (c) the output of the SBCI.

The method of calculating the TP rate is shown in Figure 4. In Figure 4(a), a sample EEG signal and in Figure 4(b), the output of the physical switch are shown. As stated earlier, data from 1 second before to 1 second after, a decision point is used for classification. Assuming the system has no processing delay, and the SBCI system has the ideal detection rate, the output of the SBCI system should be as demonstrated in Figure 4(c). In other words, the IC command is initiated by the system 1 second after pressing the switch. Although, the exact timing of the switch activation is known, the neurological phenomena may not be completely time-locked to the switch activation. As a result, we also considered any activation in the time range [−0.125 + 0.125] seconds around the expected activation of the switch as a true positive (see Figure 4(c)). The rest of activations were treated as false positives.

## 4. Results

### 4.1. Analysis of the SBCI's Performance on Artifact-Contaminated Data

Table 3 shows the averages of the TP and FP rates for both noncontaminated and artifact-contaminated data. The averages are calculated over five outer validation sets. The numbers in parentheses show the standard deviations.

To examine the effect of artifact-contamination on the performance, we carried out a two-way analysis of variance (ANOVA). First, the “TP rate” was considered as the dependant variable, and “participant” and “artifact contamination” were considered as the independent variables. As for “artifact contamination”, there were two cases: “contaminated” and “noncontaminated”. ANOVA showed a highly significant main effect of “participant” (*P* < 0.001). The main effect of “artifact contamination” was not significant (*P* > 0.05). The average TP rate over all participants was 56.2% for noncontaminated data and 51.8% for artifact-contaminated data. The average TP rate thus decreased only by 4.4% when tested on artifact-contaminated data.

Next, the “FP rate” was considered as the dependant variable, and “participant” and “artifact-contamination” were considered as the independent variables. ANOVA showed a highly significant main effect of “participant” (*P* < 10^−4^), and a highly significant main effect of “artifact contamination” (*P* < 10^−4^). The effect of the interaction of both was not significant (*P* > 0.1). The average of FP rates for all four participants for noncontaminated test sets was 0.1% and 0.4% for artifact-contaminated data. As a result, although the difference between the average FP rates was statistically significant, the average FP rate of the system was increased only by 0.3% using artifact-contaminated data.

### 4.2. Test on Data Recorded in a Subsequent Session

In [3] and in Section 4.1 above, we studied the performance of the system on data collected in the first five sessions using a nested cross-validation method. We reserved the data recorded on the last (sixth) session for this study and denoted them as data recorded in a “subsequent session”. For participants AB1, AB2, AB3, and AB4, the “subsequent session” data were, respectively, recorded on days 2, 1, 6, and 2 after recording the data used for designing the system (see Table 2).

The “subsequent session” data can be considered as a “complete” test set, as no part of these data was used for designing the system, and no part of the data (whether or not contaminated with artifacts) was rejected during the analysis.

The performance of the SBCI when tested on the “subsequent session” data is reported in Table 4. The columns represent the TP and FP rates, and the rows represent the participant IDs. Two sets of NC data were considered: the NC data collected during the NC sessions, that is, during the two-minute sessions where movements were not performed, and the NC data collected during the sessions where intentional movements were performed. For the latter data, the NC data were collected for epochs between two consecutive movement attempts.

The TP and FP rate results of the system calculated using data recorded in the first five days are also reported in Table 4. Please note that these values are the combined results of both artifact-contaminated and noncontaminated data. In [3], the five-fold nested cross-validation analysis resulted in five different sets of features and classifier parameter values for each participant. The results in Table 4 are thus shown after averaging over the five outer cross-validation sets for each participant. The numbers in parenthesis are the standard deviations.

To further examine the performance of the SBCI, we carried out a two-way ANOVA study. First, the “TP rate” was considered as the dependant variable, and “participant” and “session” were considered as the independent variables. As for “session”, there were two values: “current” and “subsequent”. We compared the TP rates attributed to classifying epochs in the “current” test set with those attributed to classifying epochs in the “subsequent session”. ANOVA showed a significant main effect of “participant” (*P* < 0.01), but it did not show a significant main effect of “session” (*P* > 0.05). The average TP rate on the current test sets was 52.7%, and the average TP rate on the data in the “subsequent session” was 48.8%.

Next, we compared the FP rates on the “current” test sets, with the FP rates of the data labeled “subsequent session” ANOVA showed a highly significant main effect of “participant” (*P* < 10^−4^), a significant main effect of “session” (*P* < 0.01), and a highly significant effect of the interaction of both (*P* < 10^−4^). The average FP rate on the “Current” test sets was 0.4% and 0.8% on the data in the “Subsequent session”.

Please note that the average TP rate over participants AB1 to AB3 was 49.8% for the first 5 sessions versus 51.9% for the subsequent session. The average FP rate was 0.4% for these 3 participants for both conditions. These results show that, with the exception of AB4, the system did maintain its performance when tested with the new data sets.

**Figure 5:**
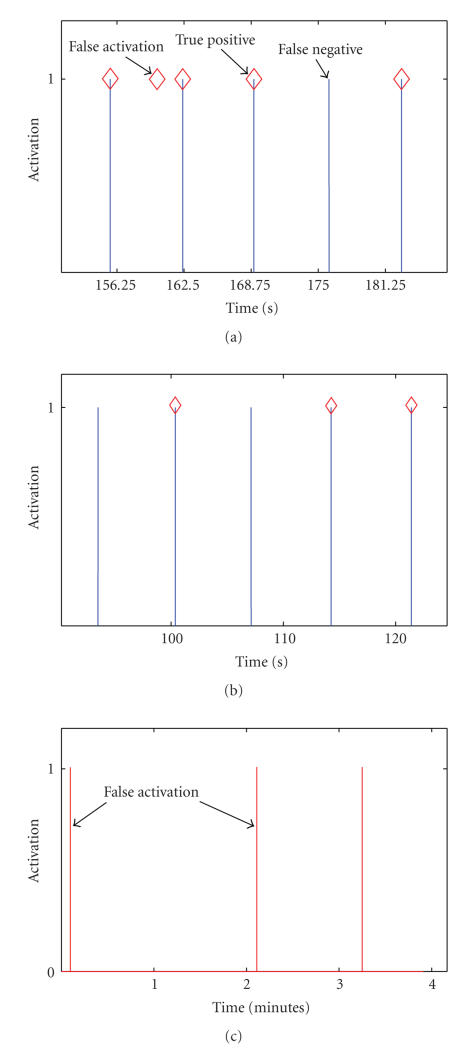
The SBCI output during periods when finger movements were executed for (a) subject AB1, (b) subject AB2, and (c) the output of the SBCI durin NC sessions when movements did not occur for subject AB.

We have plotted the output of the SBCI system for two participants to show that the detection of an IC command did coincide with the onset of the movement (see Figure 5). In order to have a clearer picture, the output is plotted for small representative time duration (around 20–30 seconds). The onset of movement is plotted as a solid line, and the output of the SBCI is plotted as a diamond. For one participant (participant AB1), TPs and FNs are also shown (see Figure 5(a)). Please note that the *x*-axis represents seconds. These results indicate that the SBCI does indeed detect the IC command, since the SBCI detects the pattern around the time of activation of the switch.

We have also shown the output of the SBCI during NC sessions for a representative user (participant AB1). For clarity, the output of the SBCI system is plotted as a solid line (see Figure 5(c)). Please note that here the *x*-axis shows minutes and not seconds. These plots show that the system is able to maintain an NC state for a long period of NC data. This is a noteworthy advantage as relatively high FP rates are known to be frustrating to users [22].

### 4.3. The Effect of Adding a Debounce Component

As discussed in Section 4.2, when the system was tested on a subsequent session, its performance deteriorated for participant 4. To decrease the FP rate, some studies have suggested the use of post-processing such as introducing the concept of refractory periods [8] or a debounce component [22]. In this section, we examine the effect of adding a debounce component to the output of the SBCI.

**Figure 6:**
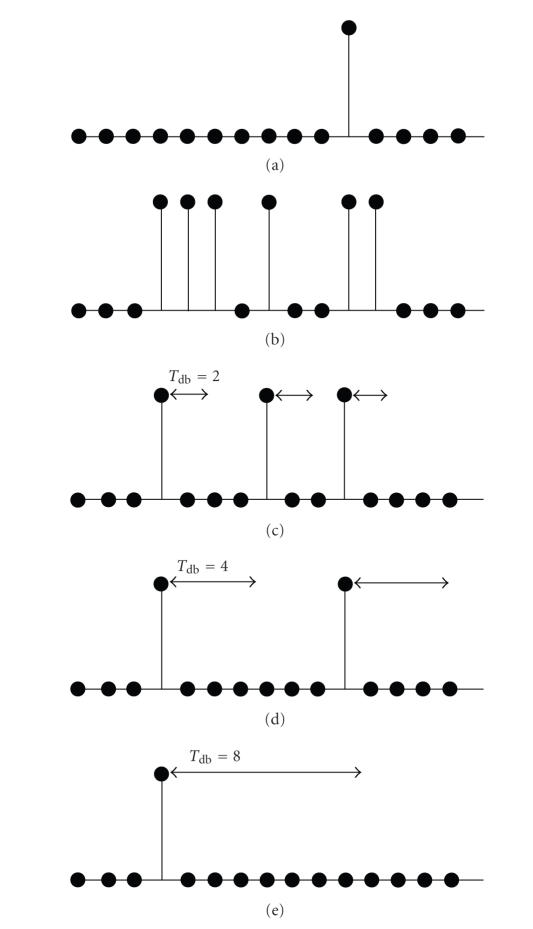
The operation of a debounce component.

Debouncing the output of an SBCI system in a manner similar to that of debouncing physical switches has been shown to improve the FP rate [22]. The debounce component continuously monitors the output. After an activation is detected (i.e., a change in state from 0 to 1), the output is automatically set to the logical state “1” (IC) for one sample. However, if a debounce window is present, then the output is forced to stay at “0” (NC) for a period of *T*_db_ samples, where *T*_db_ is the width of the debounce window base on the number of decisions. The function of a debounce component is demonstrated in Figure 6. Figure 6(a) shows the output of the switch. There is one activation towards the end of the window shown in this figure. The points marked by a black circle show the time samples. Please note that the time between two consecutive decisions (black circles) by the SBCI system is 0.125 second, as the SBCI makes 8 decisions per second [3]. Now consider the output of the SBCI as shown in Figure 6(b). There are many FPs, even though the system was able to correctly detect the IC command. Figure 6(c) shows the output of the system when a debounce component with a length of *T*_db_ = 2 decision samples is added to the system. As can be seen, some FPs are blocked by the debounce component. As the length of the debounce window increases to *T*_db_ = 4 decision samples (see Figure 6(d)), the number of FPs sharply drops. However, as Figure 6(e) shows, when the length of the debounce window is *T*_db_ = 8 decision samples, the presence of an earlier FP activation forces the output of the SBCI to have the logical value of “0” for 8 samples. Thus, the TP activation at the end of this epoch is unfortunately no longer detected. Figure 6 clearly shows that a tradeoff exists in choosing the length of the debounce window.

**Figure 7:**
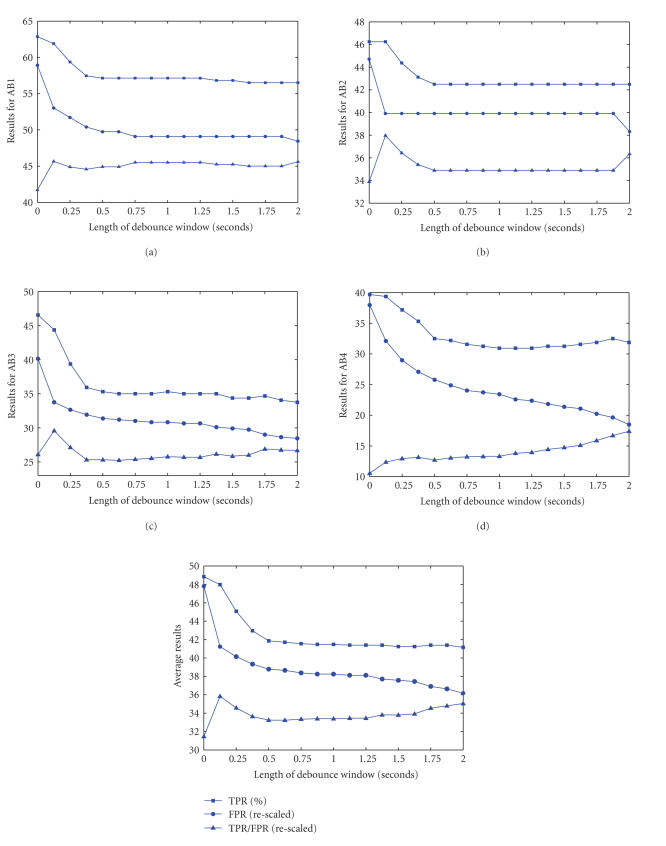
The TP rate, FP rate, and the TPR/FPR ratio as a function of the length of the debounce window for (a) subject AB1, (b) subject AB2, (c) subject AB3, (d) subject AB4, and (e) averages of all four participants.

We have calculated the performance of the SBCI system when the debounce component is added. Figures 7(a)–7(d) show the TP rate (TPR), FP rate (FPR), and TPR/FPR rates for all participants, respectively, as a function of the length of the debounce window (in seconds). Figure 7(e) shows the average plot for all participants. Please note that as the scales of TPR, FPR, and TPR/FPR are different; we had to rescale the FPR and TPR/FPR so that all plots can be shown in the same graph. As these figures show, as the length of the debounce window goes from 0 to 1 sample (0.125 second), a drop occurs in both TP and FP rates (especially a larger drop in the FP rates). As a result, the TPR/FPR ratio increases for all participants. These results indicate that a very small debounce window (*T*_db_ = 0.125 second) had a positive effect on the performance of the SBCI system. However, a further increase in the length of the debounce component did not increase the TPR/FPR ratio by more than the value achieved by *T*_db_ = 1 decision sample (or 0.125 second). This is the case with the exception of participant AB4, where a larger debounce window improved the performance. Thus, we conclude that in this study a minimal debounce window yielded superior results for most participants, however, a customized debounce window would further improve the performance of some individuals.

## 5. Conclusions and Discussion

Developing a new BCI system consists of two stages: designing the system and further testing its performance. The second stage involves a wider exploration of the performance of the system so as to investigate how well it performs under different situations including online testing using data from individuals with motor disabilities and the stability of the system's performance over time. Another important test involves evaluating the system's performance over data contaminated with artifacts. This is because in online applications artifacts occur frequently. As a result, it is necessary that the system handles the artifacts efficiently.

One approach for handling artifacts which has not yet received attention in the BCI literature is to design a BCI system that is robust in the presence of artifacts. One solution that increases the robustness to artifacts is that the BCI system employs more than one neurological phenomenon. In this paper, we investigated how much the performance of an SBCI system that uses more than neurological phenomenon (each belonging to a different frequency band) would be affected by the presence of artifacts. For this purpose, we carried out two studies to further explore the performance of an SBCI system that uses three specific neurological phenomena [3]. Specifically, we analyzed its performance on data contaminated with large-amplitude eye-movement artifacts and on data recorded in a subsequent session. Furthermore, we analyzed the effect of adding a debounce component to the output of the BCI system.

The results of our analysis show that the average TP rate does not change much when periods with EOG artifacts are rejected compared to the case when they are present in the test set.The average TP rate dropped only by 4.4% when artifact-contaminated data were used (from 56.2% to 51.8%). Further evidence shows that large-amplitude eye-movement artifacts do not have a great impact on the detection capability of this system. This robustness in the performance can be attributed mainly to the use of three neurological phenomena from different frequency bands for detecting the intentional control (IC) pattern.

Although the average FP rate increased from 0.1% to 0.4%, this value remains lower than the FP rates of some of the recently developed EEG-based SBCI systems with the same (or higher) output rates and at the relatively same TP rate [7, 9, 22–24]. This means that, on average, this SBCI system generates lower error rates compared to these systems.This change in the FP rate is a tradeoff as the system becomes available at all times, that is, even when artifacts such as eye blinks occur. While the performance of the SBCI system was better when tested on artifact-free data [3], it is only available during those time intervals for which large-amplitude eye movement activities such as eye blinks do not occur. Thus, by accepting a moderate decrease in the performance, the system becomes available for control at all times.

It should also be noted that the performance of three of the four participants did not change much when tested on the data of a subsequent session in a pseudo-online environment (see Table 4). This forms preliminary evidence that the system's performance remains robust although event-related potentials may change over time [25–27]. More research, however, is needed to verify the stability of the system's performance over long time periods.

**Figure 8:**
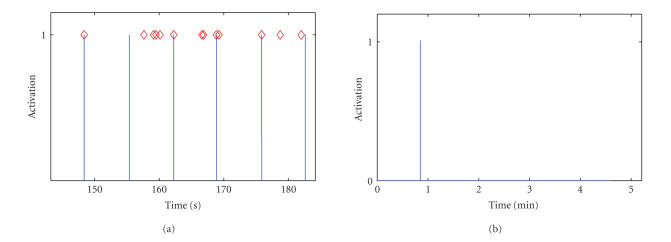
(a) The output of the SBCI during periods when finger movements were executed for subject AB4; (b) the output of the SBCI during NC sessions when movements did not occur for subject AB4.

As for participant AB4, we have observed the occurrence of many false positives between successive movement attempts (see Figure 8(a)), but during NC sessions the FP rate was very low (i.e., FP rate < 0.1%, see Figure 8(b)). We believe the reason why the system had so many FPs between different IC commands is due to the fact that the NC data between the movement attempts were not used for training the system. These observations raise the issue concerning the nature of NC data that should be used to optimally train an SBCI system. This investigation is left for future studies.

Adding a debounce component results in a decrease in the FP rates, since it masks multiple consecutive FPs. Our study shows that a small debounce window is needed for most participants for improving the performance. The optimal size of this debounce window (which was found to be equal to only one output sample) indicates that there were occasions where the system mistakenly identified two consecutive NC epochs as an IC. By adding a debounce window, multiple consecutive FPs are treated as one FP, thus the FP rate decreases. Two problems arise when with using a larger debounce window. First, if the width of the debounce window is relatively large (e.g., two seconds, as recommended in [22]), and if an FP occurs closely prior to an IC command, the IC command is blocked by the debounce component. The TP rate of the system thus drops (see Figure 6(e)). Second, as the width of the debounce window increases, periods for which the SBCI system becomes unavailable grow (see Figures 6(d) and 6(e)). This limits the practicality of the SBCI, as there will be long periods in which the system is not available. Our analysis also shows that for the proposed SBCI, using a very short duration of debounce window improves its performance in general. Wider debounce windows, however, might be needed for an individual when multiple FPs occur consecutively (e.g., participant AB4, see Figure 8(a)).

Several studies have shown that the neurological phenomena related to attempted movements by able-bodied individuals bear many similarities with those attempted by people with spinal-cord injury [21, 28–31]. Based on these studies, attempted movements from both groups activate similar cortical areas and generate similar movement patterns. This evidence has enabled us to base our analysis on the data of able-bodied subjects, who have actually executed the particular movement. By using the data of these individuals, it is then possible to label epochs of EEG signals using the output of the finger switch. The analysis of data generated by individuals with motor disabilities, however, is also of great importance and is left to future studies.

An important future study is the online testing of the system. Because of the high FP rates of other existing SBCI systems, so far only two SBCI studies have been conducted under specific conditions in an online fashion [32, 33] Since the proposed SBCI has much lower false positives than other EEG-based SBCI systems and has a good performance over artifact-contaminated periods, future research should focus on the online testing of the performance of the system.

## Figures and Tables

**Table 1:** Comparison of the TPR and FAR rates achieved in different SBCI studies.

Study	Bibliography		Frequency (number of classifier's decisions per second)	TPR(%)	FPR(%)	FAR(%)
Study 1		LF-ASD	16	<20%	2	33
	[6]	OPM		<10%		
		Mu-ASD		<10%		

Study 2	[7]		25	30%	2	25
Study 3	[8]		?	<20%	2	?
Study 4	[9]		16	67.8	2	33
Study 5	[10]		16	54.0	1	16
Study 6	[3]		8	56.2	0.1	1.2

**Table 2:** The time schedule of recording the data. For each participant, Day 1 is the first day that a participant attended the experiments. The rest of days are numbered with respect to Day 1 of that particular participant.

Participant ID	1st session	2nd session	3rd session	4th session	5th session	6th session
AB1	Day 1	Day 3	Day 5	Day 8	Day 10	Day **12**
AB2	Day 1	Day 3	Day 4	Day 8	Day 9	Day **10 **
AB3	Day 1	Day 2	Day 4	Day 8	Day 9	Day **15**
AB4	Day 1	Day 3	Day 5	Day 8	Day 10	Day **12**

**Table 3:** Comparison of the average test results on artifact-contaminated and noncontaminated data. The averages are calculated over 5 outer validation sets. The numbers in the parentheses indicate standard deviations.

Participant IDs	Test on noncontaminated data		Test on contaminated data	
	TPR(%)	FPR(%)	TPR(%)	FPR(%)
AB1	58.6			
	(8.6)	0.1	47.7	
		(0.1)	(7.9)	0.5
				(0.3)

AB2	64.2			
	(7.5)	0.0	51.0	
		(0.0)	(4.0)	0.1
				(0.0)

AB3	46.9	0.3	43.7	0.7
	(10.4)	(0.2)	(4.8)	(0.2)

AB4	55.0	0.1	64.7	0.4
	(5.3)	(0.1)	(5.7)	(0.1)

Mean	56.2	0.1	51.8	0.4
	(7.2)	(0.1)	(9.1)	(0.1)

**Table 4:** Comparison of the average results using data recorded in the first five sessions with those using data recorded in a subsequent session. The averages are calculated over 5 outer validation sets. The numbers in the parentheses indicate standard deviations.

Participant IDs	Combined test results on the first five session		Test results on the subsequentsession	
	TPR(%)	FPR(%)	TPR(%)	FPR(%)
AB1	50.3	0.5	62.9	0.3
	(8.0)	(0.2)	(8.8)	(0.2)
AB2	55.0	0.1	46.3	0.1
	(5.0)	(0.0)	(5.1)	(0.1)
AB3	44.2	0.7	46.6	0.8
	(5.7)	(0.2)	(6.0)	(0.2)
AB4	61.2	0.4	39.7	1.8
	(5.3)	(0.1)	(8.0)	(1.0)
Mean	52.7	0.4	48.8	0.8
	(7.2)	(0.2)	(9.9)	(0.7)
